# Intersubjetividad y modos de cuidado en salud: sensibilidad eco-etno-social y competencia tecnológica crítica para la calidad-equidad en la salud digital

**DOI:** 10.18294/sc.2025.5763

**Published:** 2025-07-30

**Authors:** Naomar de Almeida-Filho, José Ricardo de Carvalho Mesquita Ayres

**Affiliations:** 1 PhD en Epidemiología. Profesor titular jubilado, Instituto de Saúde Coletiva, Universidade Federal da Bahia. Profesor visitante, Instituto de Estudos Avançados, Universidade de São Paulo. Consultor Senior, Secretaria de Informação e Saúde Digital, Ministério da Saúde, Brasil. naomaralmeida@gmail.com Universidade Federal da Bahia Instituto de Saúde Coletiva Universidade Federal da Bahia Brasil Brazil naomaralmeida@gmail.com; 2 Doctor en Medicina. Libre-docencia. Profesor titular, Departamento de Medicina Preventiva, Faculdade de Medicina, Universidade de São Paulo, São Paulo, Brasil. jrcayres@usp.br Universidade de São Paulo Departamento de Medicina Preventiva Faculdade de Medicina Universidade de São Paulo São Paulo Brazil jrcayres@usp.br

**Keywords:** Salud Digital, Competencia Tecnológica Crítica, Equidad en Salud, Cuidado Integral Digital, Digital Health, Critical Technological Competence, Health Equity, Comprehensive Digital Care

## Abstract

En este ensayo, proponemos que la teoría del proceso de trabajo en salud de Mendes Gonçalves puede contribuir a una teoría crítica de la salud digital capaz de deconstruir la alienación tecnológica en los procesos de transformación digital en el área de la salud. En primer lugar, se identifican modos de cuidado y modelos de organización tecnológica de la atención sanitaria. En segundo lugar, analizamos el modo de cuidado colectivo-informacional orientado a las redes sociotécnicas de salud, destacando las prácticas colaborativas arraigadas en los territorios y las realidades locales. En tercer lugar, se propone una plataforma teórica para la comprensión de la transformación digital en salud, a partir de la articulación de los siguientes conceptos: apropiación sociotécnica, metapresencia, competencia tecnológica crítica, calidad-equidad, y sensibilidad eco-etno-social. Finalmente, esbozamos la fundamentación epistemológica y teórica necesaria para una “epistemodiversidad digital”, entendida como una ecología de saberes, en la que los objetos técnicos, los sistemas sociotécnicos, los agentes humanos y los seres transhumanos puedan coexistir, interactuar y producir cambios sistémicos en el campo de la salud, con el protagonismo de trabajadores y trabajadoras de la salud y una amplia participación popular.

## Abstract (en)

In this essay, we propose that Mendes Gonçalves’ theory of the labor process in health can contribute to a critical theory of digital health capable of deconstructing technological alienation within digital transformation processes in the health sector. First, we identify modes of care and models of technological organization of healthcare delivery. Second, we analyze the collective-informational mode of care oriented toward sociotechnical health networks, highlighting collaborative practices rooted in territories and local realities. Third, we propose a theoretical platform for understanding digital transformation in health, based on the articulation of the following concepts: sociotechnical appropriation, metapresence, critical technological competence, quality-equity, and eco-ethno-social sensitivity. Finally, we outline the epistemological and theoretical foundations necessary for a “digital epistemodiversity,” understood as an ecology of knowledges in which technical objects, sociotechnical systems, human agents, and transhuman beings may coexist, interact, and produce systemic changes in the health field, with the protagonism of healthcare workers and broad popular participation.

## INTRODUCCIÓN

“La asistencia médica individual amenaza con transformarse en una especie de pesadilla prometeica, en la que cada nueva necesidad atendida se multiplica en otras necesidades por atender, en una escala asociada a la crisis de costos, pero aún con la amenaza de un descrédito creciente y la amenaza aún peor de su cuestionamiento como respuesta social legítima a problemas generados en la propia dinámica social, pero que no deben ser concebidos así por razones obvias”. Mendes-Gonçalves^1^

Ricardo Bruno Mendes-Gonçalves (1946-1996), médico y filósofo brasileño, uno de los íconos del pensamiento crítico sobre salud-enfermedad-cuidado en América Latina, desarrolló la *teoría del proceso de trabajo en salud*^1^, que partió de la crítica marxista y fue renovándose a través de los discípulos e interlocutores de Mendes-Gonçalves, en diálogo con autores más o menos relacionados con esta tradición, como los teóricos frankfurtianos de tres generaciones (Adorno, Horkheimer, Marcuse, Benjamin, Habermas y Honneth), con la tradición fenomenológica que desemboca en la hermenéutica contemporánea (Heidegger, Gadamer y Ricoeur), y con la epistemología histórica francesa de la primera mitad del siglo XX, como Bachelard y Canguilhem, y posteriormente Foucault.

Sin duda, el diálogo con la obra foucaultiana ya estaba presente en la fundamentación conceptual-epistemológica de la salud colectiva, inicialmente a través de Sérgio Arouca, y luego enriquecido con las contribuciones de Cecília Donnangelo y Mendes-Gonçalves, aunque Michel Foucault continuó reformulando su tesis arqueogenealógica sobre el desvanecimiento del sujeto moderno. Sin embargo, Foucault falleció en 1984 y no llegó a presenciar los desarrollos de su provocación al analizar la emergencia de las sociedades de control, tanto en el plano general de la biopolítica como en planos particulares, como el campo de la salud.

Uno de sus principales herederos intelectuales, Gilles Deleuze, dictó tres cursos en el Collège de France entre 1985 y 1986, reunidos en un libro publicado en 1988 y en un breve artículo escrito en 1989, titulado “Post-scriptum sobre las sociedades de control”*.* En esos textos, Deleuze^2^ retoma de la biopolítica foucaultiana la proposición de que, en el cambio de siglo XIX, las sociedades de soberanía, provenientes directamente del modo de vida feudal y clásico, comenzaron una transición para convertirse en sociedades disciplinarias, alcanzando su auge en la primera mitad del siglo XX. Agrega la hipótesis de que, en el contexto de profundas mutaciones del capitalismo derivadas de las máquinas informáticas, las sociedades disciplinarias están siendo sustituidas por sociedades de control. En sus palabras, Deleuze señala:

“Las antiguas sociedades de soberanía manejaban máquinas simples, palancas, poleas, relojes; pero las sociedades disciplinarias recientes tenían como equipamiento máquinas energéticas, con el peligro pasivo de la entropía y el peligro activo del sabotaje; las sociedades de control operan con máquinas de una tercera especie, máquinas informáticas y computadoras, cuyo peligro pasivo es la interferencia, y el activo, la piratería y la introducción de virus”.^2^

En las sociedades disciplinarias hay distintos dispositivos de encierro o internados, mientras que en las sociedades de control hay diversos modos de control, que bien podríamos llamar “*controlatos*”: mientras el lenguaje común a los medios de confinamiento de las sociedades disciplinarias es analógico, el sistema de modos de control configura un lenguaje numérico (no necesariamente digital). Deleuze completa su hipótesis indicando que el lenguaje numérico del control está compuesto por cifras, que determinan el acceso a la información. Los binarios colectivo-individual, persona-sociedad, masa-individuo pierden sentido porque, en ese proceso, las masas se convierten en muestras, poblaciones, mercados o “bases de datos”, y los individuos se transforman en nuevos sujetos sociales, divisibles, “dividuales”.

En el posfacio a la biopolítica de Foucault, Deleuze afirma que este nuevo ser ya no es un individuo (por tanto, no divisible), sino *dividuos* (plurales y divididos). La idea del *dividuo* se recupera así en la indicación de que ese sujeto informacional que estaba siendo gestado comprende híbridos y transhumanos; es decir, se está superando la etapa del humano materialmente constituido e ideológicamente instituido. Deleuze aplica esta concepción al campo de la salud, refiriéndose a una “nueva medicina *sin médico ni paciente*”, que rescata enfermos potenciales y sujetos en riesgo, lo cual de ningún modo representa un progreso hacia la individuación, como suele afirmarse, sino que sustituye el cuerpo individual o numérico por la cifra de una materia “dividual” a ser controlada.

En estos más de treinta años desde que Mendes-Gonçalves elaboró su teoría de las tecnologías en salud, la intensificación tecnológica se ha acelerado, revelando contradicciones en la construcción de sentidos y prácticas que necesitan ser identificadas e investigadas. Las transformaciones en el conocimiento biológico y sus repercusiones en la configuración de las prácticas de salud, por un lado, intensifican las características de un modelo biomédico que incorpora progresivamente tecnología, prioriza la especificación de elementos para medicar y, al mismo tiempo, regular y controlar; en la lógica de una prevención que interviene cada vez más en la cultura y en la vida cotidiana. Por otro lado, la cuestión de la tecnología y de la práctica médica necesita hoy ser repensada desde otro aspecto: las tecnologías de la información y los consecuentes cambios introducidos en la vida social y en las formas de apropiación del conocimiento científico, ampliando el interés y la demanda social por estos saberes, técnicas y tecnologías^3^. Es decir, la teoría crítica de las tecnologías en salud necesita ser revisada como una teoría crítica de la transformación digital en salud.

En esta tarea, es necesario considerar algunos lineamientos ontológicos y epistemológicos:

- la hermenéutica como base filosófica, es decir, como modo de comprender el ser del humano y de construir el conocimiento sobre ese ser^4^^,^^5^;
- la dialéctica sí mismo-otro (identidad narrativa basada en la dialéctica entre mismidad e ipseidad) como base de la individuación en y a partir de la sociabilidad y como horizonte de una mirada ética, entendida como la búsqueda de la “*vida buena* con y para otros en instituciones justas”^6^;
- una epistemología de la reflexividad, considerando la producción de conocimientos, saberes, prácticas y técnicas como una acción ético-política que construye múltiples planos de realidad^7^;
- el reconocimiento intersubjetivo basado en el amor, los derechos y la estima social como una gramática moral para la constitución de sujetos políticos^8^^,^^9^;
- la perspectiva decolonial, la pluralidad de saberes y el diálogo simétrico y reconstructivo entre diversas epistemologías como búsqueda de acceso al *buen vivir*^10^^,^^11^^,^^12^^,^^13^^,^^14^^,^^15^.

Asumimos como presupuesto que la incorporación de las tecnologías digitales en la vida cotidiana actual constituye un proceso histórico irreversible y que, al incidir sobre nuevas configuraciones de sujetos humanos “dividualizados”, tales tecnologías pasan a integrar la experiencia práctica sobre la cual, y por medio de la cual, es necesario detenerse críticamente en un proceder hermenéutico. Así, en un escenario sociopolítico y cultural modelado por las tecnologías digitales de información y comunicación en salud (TDICS), tres aspectos resultan cruciales para la reconstrucción crítica de los saberes y prácticas en salud:

- *Hermenéutica*: ¿Sigue siendo posible comprender al yo, al otro y al mundo mediante fusiones de horizontes, incluso cuando las interacciones están mediadas por tecnologías que tienden a la superficialidad narrativa (imágenes puntuales, lenguajes reducidos, identidades fragmentadas)?
- *Dialéctica de sí mismo-otro*: ¿Siguen siendo aprehensibles las dimensiones intersubjetivas del cuidado cuando este se encuentra mediado por tecnologías que dependen de la estandarización y generalización de datos?
- *Reconocimiento intersubjetivo*: ¿Sigue siendo posible mantener relaciones de cuidado fundadas en el soporte psicoafectivo, la promoción y protección de derechos, y la valorización de las singularidades y diversidades, cuando la intervención se realiza a través de protocolos y sistemas automatizados?

La pregunta “¿sigue siendo posible?” conlleva escepticismo y esperanza^16^. Las amenazas identificadas y las soluciones propuestas aún no se concretan plenamente; muchas son apuestas y proyecciones. La reflexión se centra en la automatización de interacciones previamente basadas en relaciones singulares, ahora sustituidas por algoritmos conversacionales. La cuestión central es cómo formular una hermenéutica ajustada a la infosfera contemporánea^17^, especialmente frente a la emergencia de nuevos sujetos como gemelos digitales, ciborgs e infoviduos. Esta cuestión remite a la formación de nuevos sujetos en la era digital, marcada por una biopolítica de los cuerpos informatizados^18^. El cuestionamiento fundamental es, en definitiva, cómo seguir apostando por la construcción de una salud verdaderamente colectiva, incluso cuando el horizonte normativo de una salud solidaria y efectivamente emancipadora tiene tan alteradas sus bases de operación concreta por la emergencia de las tecnologías digitales.

En este ensayo, proponemos que la teoría del proceso de trabajo en salud abre perspectivas ricas y diversas para una teoría crítica de la salud digital, capaz de contrarrestar los riesgos de la alienación tecnológica tendencial en los procesos de transformación digital en el campo de la salud, sobre todo a través de la formación de sujetos epistémicos sensibles, competentes y efectivos. Para ello, en primer lugar, buscaremos mostrar la pertinencia y vigencia de la teoría del proceso de trabajo en salud, identificando modos de cuidado y modelos de organización tecnológica de la atención en salud. En segundo lugar, analizamos el modo de cuidado colectivo-informacional orientado a redes sociotécnicas de cuidado, valorizando prácticas colaborativas enraizadas en territorios y realidades locales, desplegado en tres modelos principales: el modelo socioepidemiológico de los sistemas regionalizados de salud, el modelo del cuidado integral digital y los modelos multimodales de los ecosistemas digitales. En tercer lugar, exploramos una plataforma teórica potencialmente útil para la comprensión de la transformación digital en salud, basada en la articulación de los siguientes conceptos: apropiación sociotécnica; metapresencialidad; competencia tecnológica crítica; calidad-equidad; y sensibilidad eco-etno-social. Finalmente, presentamos el esbozo de una fundamentación epistemológica y teórica necesaria para una ecología de saberes sensibles, articulados en una “epistemodiversidad digital”, en la cual objetos técnicos, sistemas sociotécnicos, agentes humanos y seres transhumanos puedan convivir, interactuar y producir cambios sistémicos en el campo de la salud, con protagonismo de trabajadoras y trabajadores de salud y amplia participación popular.

## VIGENCIA DE LA TEORÍA DEL PROCESO DE TRABAJO EN SALUD

La vigencia del pensamiento de Ricardo Bruno Mendes-Gonçalves tiene como horizonte el sentido normativo de reconstrucción de saberes y prácticas con capacidad para promover el diálogo entre lo individual y lo colectivo en las acciones de salud -ya sea desde el diagnóstico de necesidades hasta las formas posibles de intervención- de forma dialógica (no esencialista, ni fundacionalista, ni tecnicista) y, en la medida de lo posible, emancipadora. Las relecturas de la *teoría del proceso de trabajo en salud* permiten una crítica a la praxis de la salud que, tanto desde la perspectiva de la práctica teórica como de la práctica técnica, busca activamente la transición filosófica del llamado paradigma de la conciencia, o del sujeto, hacia el paradigma de la intersubjetividad^19^, en sus implicancias ontológicas, epistemológicas y éticas. En esta línea, ya sea en la comprensión del conocimiento producido por las ciencias de la salud -en particular, los estudios sobre la epidemiología y el concepto de riesgo^20^^,^^21^^,^^22^^,^^23^-, o en la preocupación por prácticas de salud sensibles a las implicancias sociales de los procesos de enfermar y de atención a la salud^24^, avanzamos algunas proposiciones en torno a los conceptos de *vulnerabilidad*, *cuidado* y *calidad-equidad*.

Al analizar la historicidad de las prácticas de salud, Mendes-Gonçalves^1^ identifica dos configuraciones tecnológicas polares en el modo de producción/operación de sus saberes en las sociedades occidentales contemporáneas -entendiéndose polo en sentido dialéctico, es decir, como teniendo cada uno una configuración intrínsecamente dependiente de su opuesto y, al mismo tiempo, negándole algunos de sus aspectos fundamentales. En este sentido, describe los modelos *clinicopatológico* y *epidemiológico* de organización tecnológica, destacando sus diferencias sobre la base de binarios conceptuales (colectivo vs. individual, social vs. biológico, histórico vs. natural), subrayando que deben ser comprendidos como matrices de conceptos, recursos intelectuales con los que es posible explicar la realidad concreta e intervenir en ella con racionalidad técnica. Para Mendes-Gonçalves^1^, a partir de estas dos dimensiones generales del objeto de trabajo de las profesiones de la salud -la dimensión individual-biológica y la colectiva-social- se han desarrollado y utilizado tecnologías médicas. Las primeras prácticas de salud del capitalismo utilizaban un concepto colectivo de “enfermedad”, en lugar de individual, creando una institucionalización complementaria de ambos modelos, basada en la noción de policía (y política) médica. No obstante, el modelo basado en la concepción de la “enfermedad” como fenómeno transindividual fue superado por el modo de organización tecnológica basado en la concepción instrumental de la “enfermedad” propia de la clínica anatomopatológica, dando lugar a una “medicina tecnológica” coherente con las necesidades del capitalismo emergente.

Partiendo de la proposición general elaborada por Mendes-Gonçalves^1^ del trabajo en salud como práctica técnica y práctica social, y ampliándola al distinguir entre “modos de cuidado” y “modelos de organización de la atención en salud”, proponemos que, en un enfoque tipo-ideal, existirían dos modos de cuidado en salud: el modo individual-etiológico y el modo colectivo-informacional. Cada uno de estos modos de cuidado se despliega en diferentes conjuntos de estrategias y prácticas que se expresan como modelos de organización de la atención en salud. La Tabla 1 esquematiza esta tipología de modos y modelos de cuidado-atención en salud.

**Tabla 1:** Tipologia de modos de cuidado e modelos de atenção em saúde.

Modos de cuidado	Formas de organización de la atención en salud
Individual-etiológico	Modelo clínico-semiológico de cuidado artesanal
	Modelo biotecnológico intensivo en tecnología
Colectivo-informacional	Modelo socioepidemiológico reterritorializado
	Modelo de “cuidado integral digital”
	Modelos multimodales de ecosistemas digitales

Fuente: Elaboración propia.

A pesar de los posibles beneficios que pueden traer las tecnologías digitales de información y comunicación en salud (TDICS) con relación a la ampliación del acceso y en el aumento de la autonomía de las personas respecto de su salud, en las últimas décadas se ha consolidado en muchos países una perspectiva individualista, tecnicista y medicalizante del cuidado en salud. Proponemos aquí denominarla modo de cuidado “individual-etiológico”. Este modo de cuidado concreta una racionalidad clínica centrada en la enfermedad y en la búsqueda de causas específicas a ser eliminadas o compensadas, orientada por una lógica diagnóstica basada en indicios (signos y síntomas) y en una terapéutica quirúrgica o farmacológica generada a partir de nosologías y etiologías. Este modo opera tradicionalmente bajo la suposición de que la salud puede ser protegida o restaurada mediante la prevención y/o corrección de anomalías, lesiones o disfunciones biológicas localizadas en cuerpos individuales. Su forma clásica se despliega en dos modelos: un modelo “clínico-semiológico” de cuidado artesanal y un modelo “biotecnológico” intensivo en tecnología.

### Modelo clínico-semiológico

En el modelo clínico-semiológico, predominante hasta mediados del siglo XX y aún presente en contextos específicos de la atención primaria de salud, la práctica del cuidado se basa en la escucha del paciente, la observación sistemática, la realización de exámenes físicos y la interpretación clínica de signos y síntomas^25^. Se trata de una clínica artesanal, centrada en la relación médico-paciente y en la capacidad heurística del profesional de salud para interpretar manifestaciones singulares del sufrimiento a partir de referentes etiológicos aproximativos. En gran medida, este modelo expresa, como residuo ideológico, el *ethos* hipocrático de la medicina occidental y tiene como referencia la tradición anatomopatológica, en la cual el cuerpo enfermo puede ser descifrado mediante saberes clínicos, semiológicos y experienciales acumulados como casuística.

Producto del iluminismo del siglo XVIII y de la modernidad del siglo XIX, el modelo clínico-semiológico constituye uno de los pilares históricos de la práctica médica occidental moderna, enraizado en la tradición anatomopatológica que consolidó el cuerpo humano como objeto de la clínica^26^^,^^27^. La racionalidad de este modelo, basada en la observación directa, en la escucha atenta y en la experimentación controlada, configura un paradigma de cuidado artesanal en el que el diagnóstico se fundamenta en la identificación de signos y síntomas manifiestos, remitiendo la comprensión del objeto de intervención clínica a lesiones anatómicas, disfunciones fisiológicas y desequilibrios metabólicos.

Como subrayan diversos autores^16^^,^^25^^,^^26^^,^^27^, la clínica semiológica es una práctica interpretativa que valoriza el contexto, el gesto y el discurso, en oposición a la estandarización abstracta de las prácticas biomédicas contemporáneas. En este modelo, la consulta se entiende idealmente como un encuentro singular entre dos sujetos, mediado por instrumentos semiológicos clásicos -la anamnesis, la inspección, la palpación, la percusión, la auscultación-, pero también por vínculos afectivos y por una ética de la atención. La mirada clínico-semiológica se construye como saber artesanal, en el que la experiencia del profesional y la narrativa del paciente se entrelazan, dando origen a una comprensión compartida del proceso de enfermar. La clínica se presenta aquí como arte y técnica: exige del profesional la capacidad de escuchar, examinar e interpretar de forma sensible las manifestaciones del sufrimiento humano. La clínica artesanal comprende, en ese sentido, un espacio de acogida y reconocimiento de las singularidades, aunque en la práctica sufra interferencias nada despreciables provenientes de las exigencias impuestas por la lógica de la productividad y por las normatividades de los algoritmos de la *medicina basada en evidencias* (MBE). No obstante, una parte importante de su potencia reside justamente en la valorización de la escucha, de la presencia, de la corporeidad y del tiempo necesario para la construcción del cuidado.

Existe una tendencia a descalificar este modelo como “arcaico” o “no científico”, cuando, en realidad, se trata de una potente alternativa frente a las formas despersonalizadas y deshumanizadas de atención. Aunque fuertemente asociado al pasado, este modelo permanece vivo en las prácticas cotidianas de la atención primaria de salud (APS), especialmente en contextos periféricos y rurales, donde los recursos tecnológicos son escasos y la centralidad del vínculo terapéutico es crucial. Su resiliencia se debe a su capacidad para promover la integralidad del cuidado, la escucha activa de las subjetividades y la construcción de confianza entre profesional y usuario. Por diversas razones y de múltiples maneras, esta forma de cuidado ha sido amenazada por numerosos procesos contemporáneos. Como ya señalaba Freidson^28^, pionero de la sociología médica, el saber clínico-semiológico ha venido siendo progresivamente sustituido desde hace tiempo por dispositivos y sistemas de vigilancia organizacional y de control gerencial y profesional, que limitan la práctica reflexiva.

Por otro lado, las versiones más actuales de este modo específico de cuidado reflejan una intensa reconfiguración técnico-científica de la clínica, correspondiente a lo que Mendes-Gonçalves^1^ y Schraiber^27^ denominan “medicina tecnológica”. Esta inflexión tecnocientífica ha sido reforzada por el uso de la historia clínica electrónica de última generación y por la creación y difusión de dispositivos digitales, con la creciente incorporación de algoritmos diagnósticos y plataformas de datos en tiempo real. Con la diseminación de redes sociales y dispositivos ultraportátiles, el sujeto ha sido convocado a convertirse en agente de su propia medicalización, asumiendo nuevas responsabilidades de vigilancia sobre riesgos biológicos y conductuales, en un contexto de individualización neoliberal de la salud humana. En este movimiento, la responsabilización individual, apoyada por narrativas de empoderamiento digital, oscurece los determinantes sociales de la salud y fragmenta el cuidado, desplazando la atención de las dinámicas colectivas y los contextos socioterritoriales hacia métricas centradas en el cuerpo biológico y en el rendimiento individual^29^.

En la base económica, la financiarización de la salud y la lógica neoliberal de gestión imponen una racionalidad cuantitativa que tiende a ignorar los aspectos subjetivos y relacionales del cuidado. La expansión de sistemas informatizados, la incorporación de protocolos clínicos rígidos y la presión por metas de desempeño reducen la autonomía profesional y desfiguran la dimensión artesanal de la clínica. En definitiva, su autonomía profesional ha sido progresivamente reducida por las dinámicas de protocolización y financiarización del cuidado, lo que conlleva la erosión de la singularidad terapéutica.

También es necesario prestar atención a la hegemonía etnocéntrica de las epistemologías y racionalidades (europea, blanca, masculina y colonial) que orienta las ciencias y tecnologías del campo de la salud, cada vez más cuestionada por grupos poblacionales social y políticamente minorizados o excluidos (mujeres, personas negras, pueblos originarios, etc.), lo cual exige innovaciones tecnológicas orientadas no solo a incrementar la eficacia o la productividad, sino también, y especialmente, a garantizar la pluralidad y la equidad.

La apuesta, por lo tanto, es que la clínica semiológica pueda encontrar en las tecnologías digitales -cuando son apropiadas con sensibilidad ética y apertura a la diversidade- formas de intensificar su capacidad de acoger, comprender y cuidar, siempre que se preserven el protagonismo del sujeto y la dimensión intersubjetiva del encuentro clínico. Así, el modelo clínico-semiológico de cuidado artesanal puede contribuir a una salud digital crítica, democrática y humanizada, especialmente en el primer nivel de atención en salud. Su mayor valor reside en la integralidad de la mirada clínica y en la construcción de vínculos terapéuticos basados en la confianza. El reconocimiento de este modelo como una forma legítima y necesaria de cuidado permite fortalecer políticas de formación profesional, de valorización del trabajo en APS y de resistencia frente a la excesiva estandarización de las prácticas de salud.

Por tanto, el modo individual-etiológico debe ser comprendido como un campo de tensiones: entre tradición e innovación, entre artesanía clínica y tecnociencia, entre subjetividad y algoritmización. Su uso ético y crítico requiere el reconocimiento de sus límites, la valorización de los saberes relacionales y la incorporación de principios de equidad, integralidad y cuidado humanizado. En ese sentido, la articulación con modelos sociotécnicos de atención en salud colectivos y ampliados se vuelve indispensable para contrarrestar los efectos del exceso de individualización de las intervenciones y de la abstracción “dividualizadora” de sus tecnologías, y así promover una práctica clínica sensible a las dimensiones históricas, sociales y culturales del proceso de enfermar humano.

En el contexto actual de la salud digital, resulta urgente una reconstrucción crítica de la clínica artesanal, a la que hemos denominado Cuidado, con mayúscula^30^, que promueva de manera emancipadora su inevitable integración sociotécnica con los nuevos dispositivos digitales, especialmente en el plano diagnóstico. La digitalización del cuidado no debe significar la sustitución de la escucha por el algoritmo, ni la confusión entre singularización e individualismo (el encuentro clínico será siempre entre sujetos y en contextos sociohistóricamente situados), sino más bien la ampliación de las posibilidades de atención sobre la base de nuevas formas de creación de vínculos, de ejercicio del respeto y de búsqueda de humanización.

### Modelo “biotecnológico”

El análisis del proceso de constitución de una “medicina tecnológica”-tal como fue realizado por Mendes-Gonçalves- es antológico, lo que justifica la larga cita que se presenta a continuación:

“...las prácticas de salud, en tanto prácticas sociales articuladas a la reproducción social en los límites del capitalismo, se desplazaron de una organización tecnológica que se basó en un inicio en un modelo analógico -etapa que perduró hasta mediados del siglo XIX- hacia una etapa posterior de predominio progresivo del modelo clinicopatológico, contemporáneo a las grandes adquisiciones instrumentales que configuraron la ‘medicina tecnológica’. ‘Medicina tecnológica’ porque el volumen, la complejidad y los costos sociales de los instrumentos de trabajo fueron tornando inviable el estilo de práctica relativamente autónomo del doctor con su maletín (en el que cabían sus principales equipos, además de los configurados en su propio cuerpo), forzando la organización de la práctica alrededor de los nuevos equipos, en el hospital, de modo análogo a lo que había ocurrido en la producción industrial. Que estas modificaciones hayan alterado al mismo tiempo el alcance y los límites de las prácticas de salud, así como la naturaleza interna de su racionalidad, que el médico contemporáneo y el conjunto de otros trabajadores articulados a él se constituyan en una realidad completamente distinta de aquellos tiempos a veces recordados con nostalgia del médico de familia (aunque fuera solo de algunas familias), eso ha sido suficientemente demostrado por los científicos sociales como para que sea más que un mero recuerdo aquí. La importancia fundamental del instrumento de trabajo (es decir, la clínica profundamente transformada, desarrollada y parcializada, junto con el hospital y los equipamientos materiales allí contenidos) en la caracterización de esta práctica es lo que justifica llamarla ‘medicina tecnológica’”.^1^

En esta forma de organización del trabajo, se imponen dos cuestiones principales, con sus contradicciones y paradojas: una crisis de costos concomitante con una crisis de efectividad^1^. Por un lado, el capital social invertido en el sector salud -ya sea público o privado- no puede superar un valor compatible con el capital social invertido en otros sectores, especialmente aquellos que generan excedente económico de forma directa. Además, tanto los intereses individuales de los productores de tecnologías como la presión social asociada a la medicalización de la sociedad, transformando las prácticas de salud, condujeron a una situación disfuncional respecto de las necesidades sociales de otros campos. Por otro lado, el modelo clínico-semiológico, a pesar de su legitimación generalizada, no ha sido capaz de reducir la magnitud relativa de los fenómenos de enfermedad. Por el contrario, sería visto como un multiplicador de dichos fenómenos. Aunque esta no sea una característica intrínseca del modelo -dado que se propone actuar sobre la enfermedad ya existente-, en las ideologías que lo estructuran desde su origen histórico, se volvió legítimo entender la enfermedad como un accidente individual posiblemente influido por factores sociales, pero que debe ser resuelto por los médicos. En algunas situaciones en que los modelos se alinean, el diagnóstico y el tratamiento individual pueden ser eficaces, llevando al control de la enfermedad a nivel colectivo.

El modelo biotecnológico, intensivo en tecnología, tal como se configuró en las últimas décadas del siglo XX, está representado típicamente por la medicina basada en la evidencia (MBE). Este modelo reformula la tradición del paradigma clínico-patológico, sustituyendo la observación artesanal por la medición automatizada, la correlación anátomo-clínica por el análisis microepidemiológico controlado de grupos de pacientes, y la interpretación subjetiva por la inferencia estadística basada en poblaciones. Sus raíces históricas se encuentran en las propuestas metodológicas de la epidemiología clínica, con dos corrientes principales: la clinimetría biomédica de Alvan Feinstein, orientada a la evaluación individual del paciente en contextos clínicos específicos a partir de datos experimentales de estudios controlados; y la vertiente más poblacional, articulada por Archie Cochrane y David Sackett, que busca orientar sistemas públicos de salud basados en evidencias robustas provenientes de ensayos clínicos aleatorizados^31^.

La medicina basada en la evidencia busca racionalizar la práctica clínica mediante la incorporación sistemática del conocimiento científico disponible, organizándolo en forma de guías clínicas y protocolos estandarizados^32^. Como señala Vianna Sobrinho^33^, la medicina basada en la evidencia tiende a transformar el juicio clínico en una operación basada en la probabilidad estadística, delegando la legitimidad de la decisión médica a una jerarquización de las evidencias científicas. La racionalidad técnica se impone sobre la experiencia vivida, y el cuidado tiende a la protocolización. Aunque la medicina basada en la evidencia está formalmente estructurada sobre tres pilares -evidencia científica, experiencia clínica y valores del paciente-, en la práctica institucional, el primer pilar se hipertrofia, tendiendo a despersonalizar el cuidado y a reducir la autonomía profesional.

La hegemonía técnico-científica de la medicina basada en la evidencia fue rápidamente capturada por sistemas de gestión en salud basados en la eficiencia económica, resultando en la medicina basada en el valor (MBV), tal como fue propuesta por Porter & Teisberg^34^. El profesional de la salud pasa a operar como ejecutor de protocolos, bajo el control de sistemas algorítmicos y estructuras corporativas de auditoría. En esta vertiente, el cuidado es gestionado con base en indicadores de costo-beneficio, sin considerar las singularidades biográficas del paciente ni los contextos sociales y culturales de la atención en salud. Se trata de una racionalidad gerencial que opera mediante la informatización del cuidado, la definición de paquetes asistenciales y la imposición de metas clínicas asociadas a la remuneración por desempeño. De este modo, la hegemonía del discurso tecnocientífico marginaliza los saberes tácitos, experienciales y narrativos que constituyen la base de una clínica semiológica sensible y políticamente consciente.

### El caso de la medicina de precisión

Como ejemplo extremo del modelo biotecnológico intensivo en tecnología, emerge una “medicina de datos”, cuyos desarrollos se expresan de forma paradigmática en la medicina de precisión personalizada (MPP). Se trata de una reconfiguración profunda de la lógica clínica tradicional, basada en la producción, integración y análisis de datos masivos provenientes de fuentes diversas y complejas, tales como bancos genómicos, informaciones proteómicas, transcriptómicas, metabolómicas, ambientales, conductuales y registros electrónicos de salud.

La medicina de precisión personalizada representa un doble movimiento: por un lado, el avance técnico-científico permite intervenciones más segmentadas y, potencialmente, más efectivas. Por otro lado, alimenta una profunda reconfiguración política y económica del campo de la salud, marcada por la mercantilización de la vida molecular, la financiarización de los datos y la concentración del poder informacional en manos de empresas que operan biobancos, algoritmos propietarios y plataformas de cuidado digital. Tal como analiza Iriart^35^, este escenario favorece una nueva forma de medicalización de la vida saludable, en la cual todos son convertidos en pacientes potenciales bajo constante vigilancia biométrica.

Con la expansión de la medicina de precisión personalizada, el modo de cuidado “individual-etiológico” se articula a nuevos procesos de tecnologización de la práctica médica, incorporando pruebas genéticas, exámenes moleculares, terapias dirigidas, sensores portables, aplicaciones de monitoreo continuo y otros objetos técnicos^36^. Según Rose^29^, se produce aquí una mutación epistemológica en la manera de definir la salud, que deja de ser la ausencia de síntomas o enfermedades para convertirse en la reducción del riesgo estadístico. La enfermedad es anticipada por cálculos probabilísticos e inferencias algorítmicas, y el cuerpo humano se convierte en un banco de datos vivo y continuo, cuyas variables son constantemente monitoreadas por aplicaciones móviles y plataformas de análisis clínico automatizado.

La promesa fundacional de esta medicina hipertecnológica es, en definitiva, la máxima personalización del cuidado, anclada en la caracterización molecular de cada cuerpo biológico y en modelos predictivos de riesgo individual^36^. Esta racionalidad, sustentada en una producción intensiva de datos, está directamente relacionada con el ascenso de la bioinformática, la investigación traslacional y la cultura algorítmica en el campo de la salud. En este modo de cuidado digitalizado, el cuerpo se vuelve objeto de una lectura continua mediante dispositivos de vigilancia biométrica, y el cuidado pasa a estar mediado por plataformas computacionales. Los datos generados son procesados por inteligencia artificial, alimentando sistemas de apoyo a la decisión clínica y algoritmos predictivos que proponen conductas terapéuticas basadas en patrones estadísticos extraídos de grandes bases de datos. La etiología clínica, en este escenario de revalorización de la investigación de laboratorio, se desplaza de la anatomía patológica visible hacia los circuitos bioinformacionales que modulan, en tiempo real, el riesgo y la respuesta terapéutica del organismo. Se trata de un proceso epistémico de objetivación, desarrollado y modelado por la bioinformática, campo interdisciplinario que condensa la biología molecular -sobre todo la genómica- con las ciencias de la computación.

Esta producción de conocimiento en salud, fundada en una lógica informacional automatizada, con una práctica clínica subordinada a intereses comerciales y con los cuerpos tratados como objetos de gobernanza algorítmica y predicción mercantil, ha sido designada como “biomedicalización digital” o medicalización algoritmizada^37^. Este enfoque está dominado por la lógica de la bioeconomía, en la que el biocapital -representado por datos genéticos y biomarcadores- se convierte en un activo estratégico para el mercado farmacéutico, para las aseguradoras y para las industrias de tecnologías en salud. La lógica del mercado, aliada a la captura de datos biomédicos por parte de corporaciones, impone una valorización de la esfera biomédica de la vida humana e inserta al cuerpo como dispositivo somático y semiológico en el circuito de extractivismo de datos y mercantilización de la información. Las tecnologías digitales no solo instrumentalizan el cuidado, sino que también normativizan conductas y reconfiguran los propios conceptos de salud y enfermedad.

Las implicancias éticas, políticas y sociales de la datificación de la medicina y del cuidado de la salud son amplias y bien conocidas. En primer lugar, la desigualdad en el acceso a tecnologías de precisión intensifica las disparidades históricas en salud, creando capas diferenciadas de ciudadanía biomédica. En segundo lugar, la opacidad de los algoritmos y la ausencia de gobernanza democrática sobre los datos generan riesgos para la privacidad, la autonomía de los sujetos y la equidad del cuidado. En tercer lugar, la reducción de la clínica a operaciones estadísticas tiende a vaciar la experiencia subjetiva del proceso de enfermar, marginando las narrativas de los pacientes en favor de la objetividad automatizada de los datos.

Por otro lado, no se desconoce que el uso ético, transparente y socialmente orientado de las tecnologías basadas en datos puede ofrecer contribuciones relevantes al cuidado, siempre que esté anclado en principios de justicia informacional, soberanía tecnológica y regulación pública. Iriart^35^ destaca que la intensificación del uso de tecnologías digitales en el cuidado individualizado promovido por la medicina de precisión personalizada refuerza la exclusión de los grupos más desfavorecidos, agudizando las desigualdades en salud, dado que el acceso a los recursos tecnológicos sigue siendo desigual.

Como reacción a este escenario de hipertrofia tecnocrática, emergen críticas a la insuficiencia epistemológica de la medicina de precisión personalizada frente a la complejidad de los contextos clínicos reales. Tales críticas abrieron espacio para enfoques que valorizan los saberes situados y la singularidad de los sujetos. Iniciativas como la de la Canada’s Drug Agency^38^, con el concepto de “evidencias del mundo real” (*real world evidence*), o la de Dipex^39^, que explora contribuciones de la investigación cualitativa a partir de narrativas de usuarios y profesionales de la salud, buscan integrar datos extraídos de la práctica cotidiana al proceso de toma de decisiones y manejo clínico, incluyendo registros electrónicos de salud, plataformas digitales, observaciones clínicas y experiencias de los usuarios y profesionales. Estas propuestas apuntan a rescatar la riqueza de los contextos vividos para ampliar y diversificar formas legítimas de producción de conocimiento clínico. Sin embargo, incluso esta idea puede ser instrumentalizada por las lógicas de control algorítmico y de financiarización de la atención si no va acompañada de mecanismos participativos y éticos de gobernanza.

El modo de cuidado “individual-etiológico”, tanto en el modelo clínico-semiológico como en las diferentes formas de una clínica intensiva en tecnología, como la medicina de precisión personalizada, puede ser comprendido como un campo ambivalente: al mismo tiempo que ofrece herramientas valiosas para ampliar la seguridad y la efectividad del cuidado, tiende a deshumanizar la atención y a restringir la autonomía de los sujetos. Un desafío contemporáneo importante será, por lo tanto, articular innovación tecnológica, saberes científicos y experiencias subjetivas de manera que se promueva una atención en salud centrada en la equidad, la dignidad y la pluralidad de los modos de vivir y enfermar.

## CONSTRUCCIÓN DEL MODO DE CUIDADO COLECTIVO-INFORMACIONAL

El modo de cuidado colectivo-informacional rompe críticamente con el paradigma biomédico clásico, proponiendo enfoques centrados en sujetos colectivos, territorios socialmente apropiados y redes sociotécnicas de cuidado. Busca superar la fragmentación de la atención mediante prácticas colaborativas, democratizadas y enraizadas en contextos locales y culturales. Como vimos en la Tabla 1, se despliega en tres modelos: el socioepidemiológico de los sistemas regionalizados de salud, el cuidado integral digital (CID) y los ecosistemas digitales multimodales.

En los modelos socioepidemiológicos, implementados en sistemas públicos, se busca ampliar la agencia colectiva sobre el cuidado y garantizar que los datos y la información estén al servicio de la ciudadanía, y no del control. El cuidado integral digital entiende el cuidado como un proceso intersubjetivo y políticamente implicado, en el que la tecnología debe estar al servicio de la escucha, la coproducción de conocimiento y la corresponsabilidad. A su vez, los ecosistemas digitales, intensivos en tecnología, articulan agenciamientos sociotécnicos plurales orientados por finalidades públicas y solidarias.

Desde esta perspectiva, la salud digital no es un conjunto neutral de herramientas, sino una *infoestructura* integrada a la vida social, con flujos de datos, plataformas y redes marcadas por dimensiones políticas, éticas y epistémicas. Tales modelos requieren gobernanza democrática, interoperabilidad, transparencia informacional e inclusión digital efectiva. La articulación entre soberanía tecnológica, participación social y alfabetización digital crítica es esencial para evitar formas de colonialidad digital, en las que los saberes locales son suprimidos en favor de epistemologías tecnocráticas y algoritmos opacos^40^. En suma, el modo colectivo-informacional propone una inflexión radical en la salud digital: datos, tecnologías y redes al servicio de prácticas de cuidado más horizontales, solidarias y democráticas, que reduzcan desigualdades, valoricen el bien común y construyan, a partir de la fusión de horizontes diversos, procesos de reconocimiento intersubjetivo guiados por el horizonte normativo del buen vivir.

### Modelo socioepidemiológico

**Figura 1:**
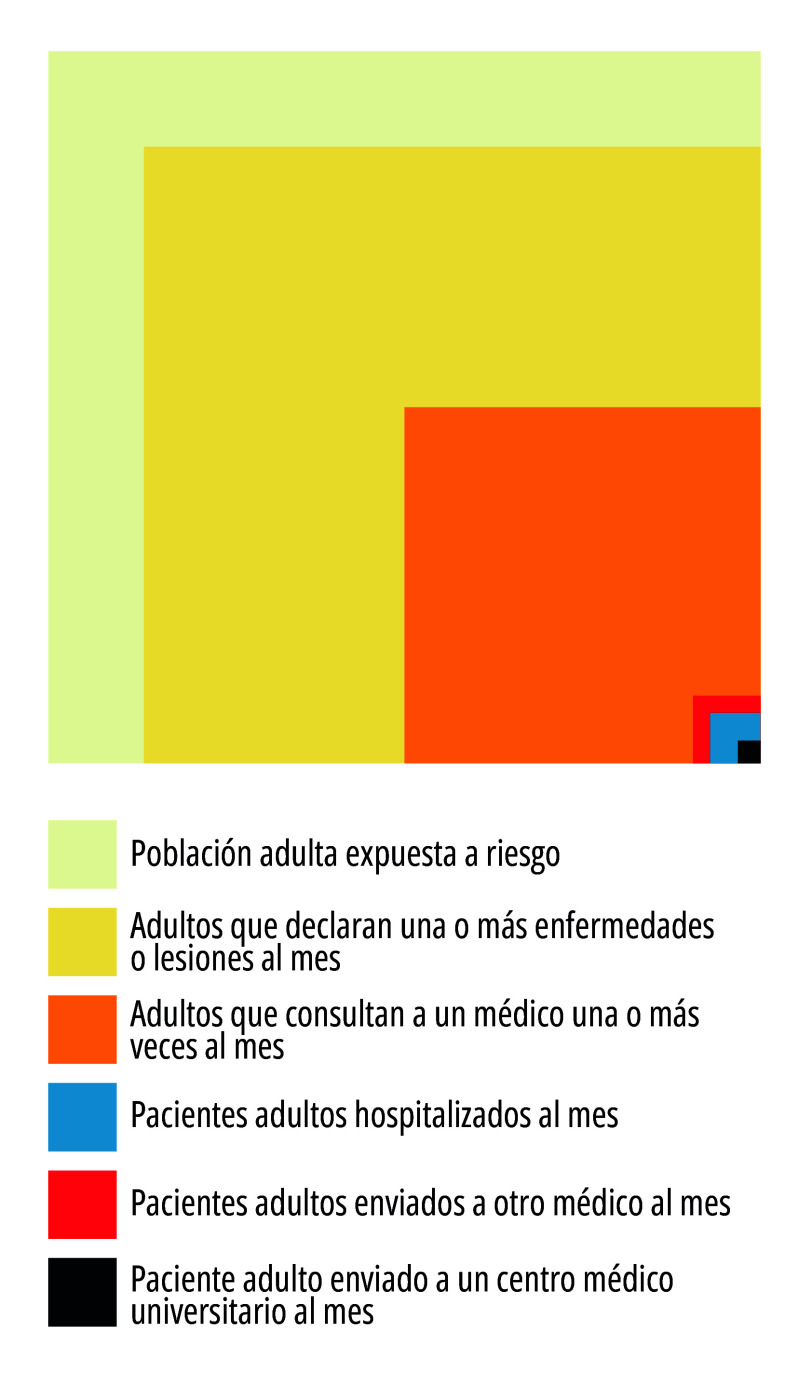
Modelo de trayectorias de búsqueda de cuidado en salud de Kerr White.

El modelo socioepidemiológico de atención en salud digital constituye un contrapunto a la lógica individualizante de la clínica tradicional, al proponer enfoques centrados en poblaciones, territorios, trayectorias de cuidado y determinantes sociales. Su principal característica es el uso de indicadores sociodemográficos y epidemiológicos para analizar patrones de salud, enfermedad y acceso a los servicios, articulando ciencia, políticas públicas y participación social. Su principal referencia lógica es el esquema de Kerr White, un marco conceptual relevante que muestra que la mayor parte de los problemas de salud no son tratados en servicios especializados (Figura 1).

Fuente: Adaptado por Spinelli et al.^41^

Con la propuesta de una “ecología del cuidado médico”, White^42^ presenta una pirámide de atención en salud, mostrando que solo una pequeña parte de la población demanda atención hospitalaria. Esto refuerza la importancia de modelos basados en información poblacional, vigilancia en salud y acciones intersectoriales, fundamentos de la salud colectiva^41^. En esa lógica, los modelos tecnológicos de cuidado seleccionan fragmentos de la realidad como atributos individuales significativos. Así, el concepto de “enfermedad” puede ser utilizado como herramienta parcial, tanto para identificar atributos como para intervenir terapéuticamente en lo colectivo a través de lo individual. Los datos epidemiológicos no se comprenden como registros neutros, sino como construcciones influenciadas por contextos políticos, económicos y culturales.

Como destacan Breilh^43^ y Ayres^30^, la producción de conocimiento y el uso de la información en salud deben considerar los perfiles de vulnerabilidad y las resistencias estructurales que moldean los modos de vivir, trabajar, enfermar y morir. En contraste con los modelos biomédicos, que tratan al individuo como una unidad aislada, el modelo socioepidemiológico comprende el proceso de enfermar como un fenómeno históricamente determinado y socialmente distribuido.

La crítica a la neutralidad de los datos es central en este modelo. La aparente objetividad de las bases de datos a menudo encubre vacíos y distorsiones que afectan a los grupos marginados. Por eso, se propone una vigilancia crítica y ética, en oposición a una vigilancia punitiva, orientada a acciones más inclusivas y eficaces. Iriart^35^ defiende una vigilancia emancipadora, sensible a las desigualdades y promotora de justicia social. Los megadatos y las plataformas pueden, así, servir a la formulación de políticas públicas orientadas al bien común, siempre que su gestión se lleve a cabo mediante procesos democráticos y ético-políticos.

En Brasil, el Sistema Único de Salud (SUS) busca realizar este modelo, integrando sistemas como el Sistema de Informação da Atenção Básica (SIAB), el SUS eletrônico (e-SUS), el Cadastro Nacional de Estabelecimentos de Saúde (CNES), el Sistema de Informação de Agravos de Notificação (SINAN), el Sistema de Informação sobre Mortalidade (SIM) y el Sistema de Informação em Saúde para a Atenção Básica (SISAB), con el objetivo de constituir bases sólidas para la gestión, evaluación y monitoreo de inequidades. La interoperabilidad entre estas bases, aliada a principios de equidad y territorialización, permite identificar exclusiones, necesidades de cuidado y uso racional de recursos. Las tecnologías digitales amplían la capacidad analítica de la epidemiología, promoviendo conexiones entre bases de datos y favoreciendo políticas públicas más sensibles y reactivas, siempre que estén acompañadas por una gobernanza participativa, transparencia, control social y soberanía sobre los datos.

En este modelo, la vigilancia debe ir más allá de la institucionalidad, siendo incorporada a la mirada comunitaria sobre los propios problemas y buscando las voces de grupos poblacionales histórica y socialmente excluidos y vulnerabilizados, especialmente por opresiones de clase, raza, género, orientación sexual y otros “desvíos” respecto de los patrones normativos dominantes. El modelo socioepidemiológico digital propone el diálogo con saberes locales y la participación ciudadana. Esto permite construir agendas de salud “desde abajo hacia arriba”, con apoyo de herramientas digitales que posibiliten la elaboración de mapas comunitarios, el monitoreo participativo de políticas y espacios deliberativos mediados por conectividad. Lo digital, así, puede redefinir las relaciones entre datos, saberes, cuidado y biopolítica. El objetivo es una salud pública fortalecida, digitalmente reestructurada, pero siempre éticamente comprometida con los derechos colectivos, la equidad territorial y la participación solidaria. Para ello, es necesario expandir los horizontes del planeamiento en salud, superando los modelos evaluativos tradicionales para reforzar los vínculos entre gestión, gobernanza y ciudadanía.

### Modelo de cuidado integral digital

El cuidado integral digital (CID) es un modelo interprofesional y transdisciplinario que busca integrar saberes, prácticas y tecnologías en un abordaje ampliado del cuidado. Inspirado en la clínica ampliada de Campos^25^, en el principio de integralidad^30^ y en el concepto reconstructivo de Cuidado^24^, esta propuesta incorpora críticamente la dimensión digital como un campo legítimo de intervención terapéutica, reconociendo los límites y los potenciales de las interacciones mediadas por tecnologías.

El cuidado integral digital parte de la complejidad de las interacciones humano-tecnológicas, tratando a los dispositivos digitales como extensiones de las relaciones de cuidado, no como sustitutos de la clínica presencial. La digitalización, aunque constituye una amenaza concreta a los procesos de efectiva individuación en la experiencia de interacción yo-otro^44^, de reconocimiento^9^ y de legitimación de la diversidad en el encuentro con la alteridad^6^, puede ser pensada como un recurso para reinventar vínculos y ampliar las posibilidades del cuidado en el siglo XXI. Así, la protección de datos, la inclusión tecnológica, la accesibilidad y la regulación ética se tornan elementos centrales de la innovación. El cuidado digital debe garantizar seguridad informacional, interoperabilidad, privacidad y autodeterminación. La equidad digital -es decir, el acceso efectivo a las tecnologías- es esencial para que la telesalud no profundice las desigualdades, especialmente en regiones remotas, donde puede representar el único canal posible de atención calificada.

El cuidado integral digital no sustituye el cuidado presencial, sino que propone expandir los modos de presencia. La telesalud debe ser algo más que una solución de emergencia: un espacio de subjetivación y convivencia, siempre que sea operada críticamente y orientada éticamente. Su consolidación requiere establecer una presencia terapéutica, incluso a distancia, articulando dimensiones simbólicas, cognitivas y emocionales. No se trata de un mero canal remoto de consultas, sino de un ecosistema interactivo de cuidado, con acogida, vínculo, escucha y humanización.

La calidad-equidad debe ser el principio estructurante de estos arreglos tecnoasistenciales. En contextos de exclusión y desigualdad, la fragmentación informacional y la vigilancia digital pueden agravar problemas de salud mental y comprometer los vínculos terapéuticos. El desafío es hacer de la innovación digital un reductor -y no un amplificador- de las inequidades. Esto requiere políticas robustas de infostructura, con conectividad segura, interoperabilidad, software público y regulación ética de los algoritmos. En el cuidado integral digital es central la noción de *metapresencialidad*^45^, que asume la posibilidad de una presencia clínica consciente y activa en los entornos digitales, siempre que existan escucha calificada, protocolos de acogida y continuidad del vínculo. No se trata simplemente de ofrecer servicios remotos, sino de crear condiciones para el cuidado en el espacio digital, respetando la diversidad, las subjetividades y las singularidades^46^.

La apropiación sociotécnica de lo digital por parte de la clínica no sustituye los modelos anteriores, pero sin duda introduce en la intimidad de los procesos de trabajo en salud nuevas necesidades y finalidades -a las que toda innovación instrumental está siempre vinculada^1^- que deben ser comprendidas, de modo que sus potenciales emancipadores, así como sus vectores conservadores y/o opresores, puedan ser críticamente y públicamente apreciados. Como telón de fondo para esa comprensión, debemos tomar como referencia el fuerte potencial que tiene lo digital para ofrecer atención a gran escala, promovida por redes que valoren tanto la personalización biomolecular como la integralidad del cuidado.

Por último, un cuidado integral digital exige que las y los profesionales de la salud desarrollen competencias digitales, relacionales y éticas orientadas a la construcción de vínculos mediados, lo que requerirá inversiones no solo en la disponibilidad de recursos tecnológicos, sino también en procesos de formación y educación permanente de los trabajadores y trabajadoras de la salud. El cuidado integral digital puede así consolidarse como un proyecto metapresencial de cuidado, con profesionales, comunidades y sujetos actuando en ecosistemas amplios, sostenibles y comprometidos con la solidaridad, la diversidad, la justicia social y la sensibilidad cultural.

### Modelo de ecosistemas digitales de salud

Los ecosistemas digitales son modelos multimodales de atención en salud compuestos por tecnologías, dispositivos, flujos informacionales y prácticas interdependientes, que conectan sujetos, instituciones, territorios y saberes. Modelos de esta naturaleza marcan un cambio significativo en la concepción y organización del cuidado. La metáfora ecológica resulta adecuada porque, a diferencia de los sistemas -compuestos por elementos semejantes e interrelacionados de forma predefinida-, los ecosistemas valorizan la diversidad y un equilibrio constantemente reconstruido y dinámico de sus partes, siempre en movimiento.

La ecología del cuidado en salud dentro de estos ecosistemas debe ser preservada. En ellos, los datos y la información no son neutros ni homogéneos: están enraizados en espacios concretos, con sujetos, disputas y materialidades en múltiples niveles. Sin embargo, muchas promesas de la salud digital se basan en la desterritorialización, la sustitución de la clínica presencial por mediación remota, la estandarización global, la reducción de la territorialidad a datos. Frente a ello, defendemos aquí una reterritorialización crítica de la salud digital, con tecnologías insertas en contextos locales, respetando culturas, saberes y relaciones comunitarias. Esta reterritorialización se asocia a la crítica foucaultiana de la biopolítica^47^ y a una perspectiva de la tecnobiopolítica desarrollada a partir de Stiegler^48^, que analiza la gobernanza ejercida por la técnica sobre la vida.

Desde un enfoque tecnobiopolítico, se comprende que los dispositivos digitales operan en la producción de modos de existencia y de gobierno de los cuerpos y de las poblaciones. La tecnobiopolítica se manifiesta, por ejemplo, cuando las aplicaciones de salud monitorean hábitos de sueño y alimentación y sugieren conductas; o cuando algoritmos clasifican a pacientes según perfiles de riesgo y priorizan atenciones con base en datos históricos. Desde esta perspectiva, las tecnologías digitales de información y comunicación en salud no son meras herramientas, sino formas de poder y de producción de subjetividades. Organizan el cuidado, estructuran el tiempo y el espacio de la atención, regulan comportamientos y constituyen normatividades.

Los modelos multimodales de cuidado rompen con la linealidad tradicional, creando arreglos dinámicos en los que distintas formas de cuidado pueden coexistir y complementarse. Incluyen historiales clínicos electrónicos, plataformas de telesalud, aplicaciones, biosensores, redes sociales, inteligencia artificial y espacios híbridos físico-digitales. No son únicamente entornos técnicos, sino espacios de disputas políticas, simbólicas y éticas. Su gobernanza debe basarse en valores públicos, participación social y control democrático. Las tecnologías libres, la soberanía informacional y la valorización de los saberes locales son fundamentales para que funcionen desde una perspectiva emancipadora de los sujetos.

Consolidar estos modelos requiere reconfigurar el trabajo en salud. Los equipos multiprofesionales deben estar preparados para actuar en entornos digitales, comprendiendo tanto el lenguaje técnico como las subjetividades y territorialidades de los usuarios. En articulación con el cuidado integral digital, es necesario desarrollar prácticas que cultiven la metapresencialidad -presencia ética y relacional mediada tecnológicamente- y afiancen el cuidado como valor central. Vianna Sobrinho^33^ destaca que estos son entornos en los que lo remoto y lo presencial, lo clínico y lo colectivo, interactúan de forma integrada, abriendo nuevas posibilidades para la atención integral. Así, formar profesionales con conciencia crítica sobre el sentido social de su trabajo, no alienados en el uso acrítico de estos nuevos instrumentos, con alfabetización digital, competencia tecnológica y ética informacional, resulta esencial.

La noción de ecosistemas digitales ofrece una clave para comprender las reconfiguraciones del cuidado, la información y la subjetividad en la era digital. Se trata de redes compuestas por plataformas, algoritmos, instituciones, prácticas sociales e infraestructuras integradas. La articulación entre los modelos de cuidado -desde el modelo individual-etiológico hasta el cuidado integral digital y el modo colectivo-informacional- debe ser crítica, situada y dialógica. Ningún modelo, por sí solo, responde a la complejidad de los contextos de salud. La potencia de los ecosistemas reside precisamente en su capacidad de conectar diferentes modos de atención, promoviendo sinergias basadas en la centralidad de los sujetos y en la responsabilidad colectiva. Esta transformación exige una regulación pública sólida, soberanía informacional, alfabetización tecnológica y fortalecimiento del control social. Más que aplicar tecnología, se trata de construir una salud socialmente enraizada, técnicamente situada y políticamente orientada por la emancipación. Si se atiende a los principios del SUS -y se reconfiguran democráticamente- los ecosistemas digitales pueden fortalecer dichos principios. Para ello, es fundamental construir una teoría crítica de la salud digital, con competencia tecnológica y sensibilidad eco-etno-social.

## CONCEPTOS PARA UNA TEORÍA CRÍTICA DE LA SALUD DIGITAL

La teoría crítica de la salud digital se concretará como un marco conceptual en la medida en que contribuya a procesos de transformación efectiva y sostenible de una situación concreta de salud. Una base teórica sólida, conformada por categorías consistentes y conceptos rigurosos, con principios ético-políticos y valores humanos, permite la concepción y realización de propuestas, planes, políticas y programas orientados a la transformación de la realidad sanitaria del país. Se trata aquí de la pertinencia -y quizás la urgencia- de una nueva ontología, con fundamentos sólidos en una epistemología que cuestione teorías simplificadoras de la realidad y de la presencia humana, en la medida en que uno de los efectos cruciales de la era digital será precisamente el borramiento de los límites físicos y de las unicidades sensoriales.

En la era digital, el mundo se torna datificado y tecnificado, reconfigurándose como un medio tecnocientífico-informacional en formaciones sociales organizadas por el modo de producción dominante^49^. Para comprender de manera sistemática y profunda las actualizaciones sustantivas de este modo de producción, se han propuesto diversos constructos, como economía informacional y capitalismo cognitivo^50^. En el capitalismo cognitivo, el excedente de valor -que en el capitalismo industrial era generado por la expropiación de la fuerza de trabajo de trabajadores y trabajadoras sometidos a la permanente explotación del valor de su mano de obra- es actualizado mediante códigos técnicos digitales. En este modo de producción prevalece una plusvalía tecnológica que, frente a la masiva digitalización del mundo contemporáneo, asume la forma de “plusvalía digital”, lo que justifica la denominación de este sistema-régimen como “capitalismo digital”.

En el capitalismo digital, el control del proceso productivo y el poder político resultante de ese control están cada vez más definidos por la propiedad intelectual sobre objetos técnicos (utensilios, equipos, dispositivos, artefactos), modos tecnológicos (protocolos, algoritmos y programas) y factores vinculados al diseño y las funcionalidades de los productos^51^. Las formas fundamentales de la alienación pasan a estar definidas por la naturaleza y densidad de la tecnología involucrada en el proceso productivo e incorporada en el producto industrial en la forma que podemos denominar “objeto técnico digital”.

Las formaciones sociales capitalistas se engendran y se reproducen mediante desigualdades estructurales inducidas y continuamente alimentadas por una ideología de consumo y obsolescencia. En la era digital, el modo de producción financiarizado y plataformizado, en el contexto de una “datificación” globalizada, produce nuevas formas de desigualdades. Estas desigualdades configuran *inequidades digitales* específicas, desde niveles diferenciales de alfabetización tecnológica hasta disparidades económicas en el acceso a la información y a las tecnologías digitales.

Con el advenimiento de la cibernética, la robótica, la miniaturización y los controles automatizados de la producción, se genera una alienación técnica que -por el hecho de que la tecnología pasa a ser propiedad intelectual de desarrolladores y patrimonio accionario de inversionistas- se convierte en “alienación tecnológica”. Además de fomentar desigualdades y alienación económica, política y tecnológica, el capitalismo cognitivo se sostiene en un régimen informacional y se reproduce en un registro de *hegemonía tecnológica*. Esto implica la instalación eficaz de una “ideología de la tecnología” como un proceso humano alienado, autonomizado, cosificado y despolitizado, por lo tanto, naturalizado, que alimenta un *colonialismo de datos* y una *colonialidad digital*^40^.

La alienación tecnológica resultante del capitalismo cognitivo puede ser superada mediante la combinación de apropiación sociotécnica y competencia tecnológica crítica. La racionalidad tecnológica, presente en proyectos políticos emancipadores, para ser efectiva tanto política como tecnológicamente, debe ser viabilizada mediante *sistemas sociotécnicos*. Esta constatación remite al concepto de *infoestructura*, considerada no solo como sustrato físico (infraestructura de instalaciones, equipamientos y redes), sino como un complejo informacional conformado por ecosistemas digitales. Los sistemas propietarios cerrados están siendo actualmente desafiados por estrategias de apertura y por parámetros de transparencia e interoperabilidad. Redes, programas y códigos pueden ser objeto de regulación como bien común, concesión de servicio público, política social y patrimonio colectivo de las sociedades, mediante políticas públicas que incidan positivamente sobre la salud y la calidad de vida^52^.

Este complejo proceso de transformación digital de los espacios económico-sociales y cultural-simbólicos, en el contexto de las tecnologías cibernéticas, repercute sobre todo en los campos sociales de las políticas públicas, como la salud^44^. No obstante, en el campo de la salud, desde un enfoque radical crítico, otra forma de integración tecnosocial -solidaria y participativa- puede generar prácticas sociales de incorporación de técnicas emancipadoras del sujeto humano^53^, en lugar de acciones conformistas y, en el límite, opresoras.

Como vimos, las desigualdades refuerzan un modelo de cuidado “clínico-semiológico”, orientado a lo individual-biológico: una “medicina tecnológica” que, en la era digital, se presenta como una clínica hipertecnológica personalizada, de sesgo elitista y generadora de nuevas formas de desigualdad que podemos denominar *inequidades digitales en salud*. Para enfrentar y superar estas inequidades digitales en salud, es necesario concebir, implementar y hacer realidad un modo solidario de producción de cuidados en salud con “calidad-equidad”^21^. Denominamos este concepto *apropiación sociotécnica*, entendido como una estrategia de enfrentamiento y superación de la alienación tecnológica mediante el desarrollo de *competencias tecnológicas críticas*. Por *competencia tecnológica crítica* nos referimos al conjunto de conocimientos y capacidades necesarios para que trabajadoras y trabajadores de la salud operen críticamente las tecnologías digitales^54^. En ese sentido, la apropiación sociotécnica crítica de tecnologías de ubicuidad, virtualización e inmersión, aprovechando las posibilidades de la telesalud y sus aplicaciones, permite viabilizar la *metapresencialidad* en los actos de cuidado en salud, para igualar el acceso a servicios y programas sanitarios^23^.

La fundamentación epistemológica y teórica de cada uno de los términos antes mencionados, aunque rigurosa y políticamente consistente, presenta un problema: desde nuestra perspectiva, seguimos inmersos en un sistema eurocéntrico de producción legitimada de conocimiento y erudición, que ha estado al servicio de las demandas cognitivas del capitalismo. Se trata, por muchas razones y en diversas dimensiones, de una nueva *colonialidad* (referencia respetuosa a Quijano) o de una *rugosidad miltoniana*^49^. Son saberes y prácticas que se “nortean” por características epistemológicas propias derivadas de la ciencia occidental, como por ejemplo la objetivación mediante métodos empíricos, el análisis lineal de datos/información, y el desarrollo de dispositivos tecnológicos y estrategias operativas concebidas para producir y controlar conexiones supuestamente necesarias entre eventos. Estas formas de aproximación necesitan ser *“sureadas”*.

En el campo de la salud, el enfrentamiento de la alienación tecnológica y de la colonialidad digital se dará de dos maneras articuladas: por un lado, a través de la vía de la *educación emancipadora*; por otro, mediante la *participación popular transformadora*. La concepción y realización de programas educativos basados en pedagogías emancipadoras será, sin duda, crucial para la formación de nuevos perfiles profesionales en el campo de la salud: sujetos epistémicos técnicamente competentes, políticamente comprometidos y socialmente responsables. Esto permitirá una participación popular organizada en movimientos sociales, conformando redes sociotécnicas que se despliegan también como *comunidades digitales* (espacios virtuales de involucramiento social) construidas colectivamente a través de prácticas formadoras de estrategias sensibles^55^.

## SALUD DIGITAL CON SENSIBILIDAD ECO-ETNO-SOCIAL

La salud digital (SD) puede situarse en el centro del análisis como una posibilidad estructurante de un nuevo modelo de cuidado tecnológicamente competente, promotor de calidad-equidad, con sensibilidad eco-etno-social. Para superar la alienación tecnológica y construir otra hegemonía -sin negar ni rechazar los avances de la tecnología industrial- es necesario dominar el conjunto de códigos técnicos en dirección a una *ecología de saberes sociotécnicos*, más allá de la propiedad industrial y del dominio legal de monopolios u oligopolios. Esta ecología de saberes sensibles se articula en una *epistemodiversidad digital*, en la que objetos técnicos, sistemas sociotécnicos, agentes humanos y seres transhumanos pueden convivir, interactuar y producir cambios sistémicos en el campo de la salud, con el protagonismo de trabajadores y trabajadoras de la salud y amplia participación popular.

Desde este punto de vista teórico que venimos desarrollando, lo que proponemos como reflexión se divide en tres aspectos interrelacionados:

1. ¿Siguen siendo adecuados los marcos o elementos conceptuales disponibles para una teoría crítica de los procesos salud-enfermedad-cuidado? ¿Qué aspectos necesitan ser reconstruidos para viabilizar saberes, prácticas y técnicas de cuidado en salud con calidad-equidad?
2. Considerando estos marcos, aun reconociendo insuficiencias y necesidades de reconstrucción, ¿cuáles son las prácticas teóricas y prácticas técnicas (de cuidado y formativas) requeridas para una praxis emancipadora en salud, que enfrente las transformaciones tecnológicas que trae la salud digital y apunte hacia la construcción de una competencia tecnológica crítica?
3. ¿Qué contribuciones pueden aportar en este sentido las nuevas teorías críticas, especialmente desde las perspectivas feministas, antirracistas y pro-diversidad sexual y de género?

A partir de esta plataforma epistemológica, proponemos una concreción conceptual, destacando los siguientes términos para la transformación digital del Sistema Único de Salud (SUS):

1. Apropiación sociotécnica
2. Competencia tecnológica crítica
3. Calidad-equidad
4. Metapresencialidad
5. Sensibilidad eco-etno-social
6. Cuidado integral digital

Modelos de planificación, gobernanza y gestión orientados a una práctica político-institucional realista, sensible y sustentable deben nutrir procesos sociales y normativos transformadores de los sistemas públicos de salud. Tales procesos habrán de inspirar horizontes institucionales, movimientos sociales y perspectivas políticas para una apropiación crítica del conjunto de saberes y prácticas actualmente denominado *salud digital*. Al promover ecosistemas digitales orientados a la reterritorialización y a la justicia social, la salud digital puede dejar de ser una simple extensión tecnológica de los servicios y convertirse en un campo de innovación institucional, democrática y ciudadana. Comprendida críticamente en su densidad técnica, política y ética, trasciende el campo de las tecnologías aplicadas e ingresa en un debate más amplio sobre los nuevos modos de habitar los territorios concretos y gobernar las poblaciones mediante dispositivos técnicos. De este modo, puede constituirse como una acción estratégica para la renovación de los sistemas públicos de salud.

La apropiación crítica de las tecnologías digitales de información y comunicación en salud (TDICS) requiere una integración basada en valores como la equidad, la solidaridad, la justicia social y la soberanía informacional. Esto implica construir *infoestructuras* que reflejen las necesidades de los territorios y de las poblaciones, respetando la diversidad cultural y las desigualdades estructurales que atraviesan el acceso a la salud y a la información. Ante este escenario, se hace necesario y urgente disputar estos territorios digitales con prácticas técnicas emancipadoras que valoren el saber local, el cuidado relacional y la diversidad cultural.

La reticularización crítica de los espacios digitales puede contribuir a formas de cuidado más sensibles a la realidad de los territorios y las colectividades. La promoción de la salud digital debe pensarse como una estrategia de *reterritorialización del cuidado*, en oposición a las lógicas de deslocalización y despolitización de la tecnología. Este movimiento exige el involucramiento de comunidades, profesionales y gestores en la construcción de sistemas que reflejen las necesidades reales de los territorios, que promuevan inclusión y justicia informacional, en un proceso que implica una acción decolonial orientada por la búsqueda de un auténtico compromiso práctico con el buen vivir común, en el sentido de una ética del amor, como propone Hooks^14^. Para ello, es necesario también -y, tal vez, sobre todo- deconstruir las onto-epistemologías que están en la base de las tecnociencias de las cuales se desprenden las tecnologías digitales. Sin ese esfuerzo, que exige un verdadero movimiento de *fusión de horizontes*^5^^,^^15^ entre los distintos habitantes de los “re-territorios” digitales, toda propuesta emancipadora no pasará de mera retórica. Herramientas como mapas digitales participativos, plataformas comunitarias de cuidado, sistemas de alerta epidemiológica sensibles al contexto y protocolos co-construidos con la población son ejemplos de cómo reconfigurar los ecosistemas digitales desde esta perspectiva hermenéutica y democrática.

Como hemos visto, los ecosistemas digitales -modelos multimodales de atención en salud- no subordinan la calidad-equidad del cuidado a las propiedades intrínsecas y funcionalidades operativas de las tecnologías digitales de información y comunicación en salud. El cuidado, en este arreglo, incorpora principios de justicia cognitiva y de ecología de saberes, reconociendo la validez de los conocimientos tradicionales, populares y territoriales, en diálogo con las prácticas técnico-científicas. Las comunidades digitales de cuidado, en ese sentido, se transforman en espacios de resistencia, creación y reconfiguración de lo común. Con apertura a la participación popular y al control social, estos modelos implican una crítica a la *alienación técnica*, concepto que denuncia el distanciamiento entre los sujetos y los dispositivos que median sus vidas. Superar esta alienación exige procesos de formación emancipadora, desarrollo de competencias tecnológicas críticas e inclusión activa de las personas usuarias en la gestión de los sistemas digitales de salud.

Para que la incorporación de la salud digital sea crítica y transformadora, debe ampliar el debate democrático sobre los usos de la información en salud. Esto incluye discutir las implicaciones de la vigilancia algorítmica, los riesgos de la mercantilización de los datos personales, las formas de gobernanza informacional y los mecanismos de regulación pública. La participación de usuarios y trabajadores de la salud en la definición de directrices, protocolos y prioridades tecnológicas es una condición indispensable para que la apropiación sea colectiva y no se concentre en especialistas o corporaciones. El fortalecimiento de canales de escucha, consulta y deliberación mediados por tecnologías digitales puede ampliar la transparencia, la corresponsabilidad y la legitimidad de las políticas públicas. Los consejos de salud, las defensorías, las plataformas comunitarias y los foros digitales son instrumentos esenciales para garantizar la presencia activa de la ciudadanía en las decisiones sobre el uso de tecnologías en salud. Sin embargo, es necesario superar los desafíos del alfabetismo digital, la accesibilidad y la autonomía informacional para que esa participación no sea meramente formal, sino efectiva y transformadora.

Podemos concluir afirmando la necesidad de resignificar el proyecto de protección social con base en los valores de emancipación, autonomía y justicia social, articulando demandas por igualdad y reconocimiento, así como enfrentando las nuevas formas de inequidad y exclusión producidas por la economía digital y la inteligencia artificial. En este escenario, cobra relevancia el concepto emergente de *salud digital* como vector de reorganización de los sistemas sanitarios y de reformulación de las prácticas de cuidado. No se trata solo de ofrecer tecnologías, sino de posibilitar el uso emancipador y ético de estas herramientas, promoviendo la autonomía de los sujetos y fortaleciendo prácticas de cuidado sensibles al contexto. Existe un fuerte riesgo de intensificación y profundización de las desigualdades e inequidades si estas herramientas son capturadas por intereses privados y utilizadas bajo una lógica mercantil. Por ello, se vuelve imprescindible que las políticas de salud digital se inscriban en un proyecto democrático y de transformación social más amplio, capaz de promover una apropiación sociotécnica soberana y emancipadora, al integrar deseos y aspiraciones colectivas en torno a la producción de lo común y a la radicalización de la democracia.
